# Cases of Rheumatism

**Published:** 1826-08

**Authors:** 

**Affiliations:** St. George's Hospital


### RHEUMATISM.

*Cases of Rheumatism*, (continued from page 12.) Treated at St. George's Hospital, by Dr. Chambers.

In our last Number, we gave some cases of diffuse rheumatism, for the purpose of illustrating a method of treatment which appears to be very efficacious in that form of disease. We subjoin examples of rheumatism affecting the synovial membranes, in which it will be seen that very different remedies were employed. Our motive for introducing these cases has been with a view of showing the broad line of distinction drawn at St. George's Hospital between the different forms of rheumatism, as regards the treatment. In one of the cases, (that of Hailstead,) it will be perceived that the two varieties ran into each other, requiring a corresponding modification of the remedies.

Two cases of metastasis are also given: one of *diffuse* rheumatism to the *heart;* the other of *bursal* rheumatism to the *brain*. These peculiarities in the part attacked by metastasis, we believe to be in conformity with observations made by Dr. Chambers, on an extensive scale. We hope, however, on a future occasion, to be able to make our readers more particularly acquainted with this gentleman's pathological views.

### III. *Case of Bursal Rheumatism, affecting the hand and knees.*

Susan Church, ætat. seventeen; single. June 14th, 1826.—Complains of pain and swelling occupying the extensor tendons of both hands, particularly the left. There is effusion into the sheaths of the tendons, and some of the joints of the fingers. Pulse 100; skin cool; tongue slightly furred; bowels natural; urine thick; catamenia regular. Ill nine months, with occasional swellings of synovial membranes, the urine being always thick at the time of the attacks.

> Haust. Piment. c. Vini Colchici, m. xx. sextis horis.
> Diæta parca.

16th.—Knees much distended; hands better.

> Hirudines x. genu sinistro. Lotio spirituosa articulis affectis.
> Repetantur alia.

26th.—The medicines have been continued, and the leeches applied to the left knee on the 17th, 18th, 21st, and 23d. Knee much better. Leech-bites ulcerated.

> Repetantur Lotio et Haust.

From this time she gradually recovered, and was discharged cured on the 3d of July.

### IV. *Case of Bursal Rheumatism, in which many joints became successively affected.*

May 17th, 1826.—Thomas Faulkener, ætat. twenty; a porter. Complains of pain and swelling of the knees, ankles, and right shoulder. These joints are much distended with an increased effusion of synovia: they are very stiff, and somewhat tender on being pressed. Pulse ninety, soft; skin quite cool; tongue furred; bowels open; urine high coloured, depositing occasionally a lateritious sediment, which he observed particularly just before this attack. The present symptoms are of five days' standing.

> Calomel, gr. v. hac et crastinâ nocte.
> Haust. Sennæ c. Vini Colchici, ʒss. omni manè.
> Hirudines xij. genu dextro, et x. sinistro. Lotio spirituosa.
> Diæta sit parcissima.

22d.—Knees much better. Right shoulder-joint more distended; the deltoid evidently much elevated by the subjacent fluid. Pulse natural; skin cool; tongue white; bowels open.

> Hirudines xij. humero dextro. Repetantur alia.

24th.—Shoulder relieved. The extensor tendons of the fingers enlarged, evidently by the effusion of fluid into their sheaths. Pulse eighty, soft; skin rather warm; tongue white in the centre, with red edges; bowels open; urine clear.

> Mist. Camph. ℥ iss.; Vini Colch. ʒss.; Magnes. Carb. ʒss. ter die.

> Repetatur Lotio spirituosa.

26th.—Right shoulder stiff. No fever.

> Empl. Canth. humero dextro. Repetantur alia.

29th.—Left shoulder painful.

Empl. Canth. humero sinistro. Repetantur alia.

June 2d.—Left wrist painful, swelled, distended, and very hot; the rest of the body cool. Pulse natural; tongue whitish.

Hirudines x. carpo dolenti.—Postea Lotio spirituosa.
Aquæ Piment. ℥ iss.; Vini Colch. m. xv.; Magnes. Sulph. ʒi.; Magnes. Carbon. ʒss. M. sumatur ter die.
Diæta sit lactea.

5th.—The left wrist free from pain: the right wrist attacked by the disease. Fluctuation perceptible in the synovial membrane. Pulse seventy-six, full; skin warm; tongue cleaner, but still furred; bowels open; urine free, clear.

V.S. ad ℥xij. Hirudines x. carpo dextro. Lotio spirituosa.
Repetantur medicamenta.

7th.—Blood buffy. Right knee has become affected. Pulse eighty, full; tongue furred; bowels open.

V.S. ad ℥ xij. Hirudines x. genu dextro cras manè.
Haust. Sennæ alterno mane, c. Vini Colchici, ʒss.—Repetantur aliâ.

8th.—Much relieved. Pulse soft; skin cool.

Repet.

12th.—Joint of right thumb swelled and painful. In other respects easy.

Hirudines ij. pollici dolenti. Repet. med.

From this time he gradually improved, but was very weak, with some œdema of the ankles. He was discharged, cured, on the 26th.

V. *Case in which the rheumatic affection assumed the bursal and diffuse forms alternately.*

January 8th, 1826.—James Hailstead, ætat. twenty-five; footman. Complains of pain in all the joints, which are distended with fluid. Pains worse at night, although on the whole he is better when warm. Pulse eighty, soft; skin cool; tongue furred; bowels open. Ill six weeks with present symptoms. Attributes the attack to exposure to cold.

Pulv. Ipecac. comp. ℈ss. omni nocte.

Haust. Salin. c. Vini Colchici, ʒss. et Magnesiæ Carbon. ʒss. ter die.

16th.—Colchicum increased on the 9th to m. xl. in each draught. Pains not relieved. Pulse rather full, soft; bowels open; tongue white.

V.S. ad ℥ xij. Repet. med.

18th.—Swelling of the hands and fore-arms more diffused. Pulse 120, sharp; skin hot, with occasional rancid perspirations; bowels open; tongue white; urine high coloured; blood buffy.

Calomel, gr. viij.; Opii, gr. iij. hac et crastinâ nocte.
Haust. Sennæ cras.

20th.—Still much pain in wrist and knees.

Rep. Calomel c. Opio omni nocte, et Haust. Sennæ alterno mane.

23d.—Pain, stiffness, and swelling better. Some sickness at stomach.

Rep. Calomel et Opium crastinâ nocte. Haust. Salin. Efferves. sextis horis.

26th.—Hands much better; severe pain in left knee; no swelling. Pulse 100, soft; tongue nearly natural; bowels open; skin warm.

Rep. Calomel et Opium hac et crastinâ nocte. Rep. Haust. Salin.

30th.—Medicines have been continued. Pains much better. Mouth sore.

Sumat Opium sine Calomel. Haust. Sennæ omni manè.
Rep. Haust. Salin. Habeat Gargarisma Aluminis.
Suffitus Aquæ calidæ partibus dolentibus.

February 8th.—Better. Sweats profusely at night.

Extract. Lactuc. gr. x. omni nocte loco Opii. Repetantur alia.

13th.—Some dysuria. Joints rather more painful, particularly the left wrist. Pulse eighty, soft; tongue white; bowels regular.

Haust. Salin. c. Vini Colchici, m. x. et Magnes. Carb. ʒss. sextis horis.
Emplast. Lyttæ carpo sinistro.
The other joints to be steamed as before.

15th.—Knuckle of the right forefinger is highly inflamed and distended. Dysuria.

Repet.

20th.—Ten grains of carbonate of soda added to the draught on the 17th. Knuckle of the right forefinger still inflamed and painful.

Hirudines ij. parti manûs dolenti. Repetantur alia.

23d.—One of the joints of the right ring-finger swelled and inflamed; knee and elbow also painful. Pulse soft; skin warm; tongue clean; bowels open; urine natural.

Decoct. Sarsaparillæ C. ℥ iij. c. Sodæ Carbonatis, gr. xv. sextis horis.
Hirudines ij. articulo digiti tumido.
Joints to be steamed as before.

March 1st.—Joint of finger again become painful.

Hirudines ij. digito.

3d.—Right shoulder painful.

Emplastrum Lyttæ humero.

Continued convalescent till April 10th, when he was sufficiently recovered to leave the hospital.

VI. *Case of Diffuse Rheumatism, with metastasis to the heart.*

October 5th, 1825.—Charlotte Yates, ætat. twenty; single: Pimlico. Rheumatic swelling, of a diffused character, of the right hand and wrist, and about the right knee: relieved by warmth. Pulse 110, soft; skin warm; bowels open from medicines; tongue white, furred. Ill seventeen days. First attacked with pain in the chest, which shifted suddenly to the present seat of the disease. Has taken, for the last two days, the following medicines:

Calomel, gr. viij.; Opii, gr. ij. omni nocte. Haust. Sennæ, omni mane.

10th.—No better.

Calomel, ℈ss.; Opii, gr. ij. bis die. Haust. Sennæ, omni mane.

12th.—Rheumatic swelling very severe on the left wrist; pulsation of carotids evidently increased. Pain and tenderness in the region of the heart. Pulse 120, small; tongue white; bowels open. Sleeps constantly.

V.S. ad ℥xij. et repetatur post horas quatuor nisi prius cessaverit palpitatio.
Calomel, gr. ij.; Pulv. Antim. gr. iij.; Pulv. Digital. gr. i. sextis horis.
Superbibendo Haust. Salin. c. Liq. Opii Sedativi, m. xx.

14th.—Blood slightly buffy. Chest relieved; pains and swellings subsided. Pulse soft, 100; tongue clean, moist; mouth not sore.

Repetantur medicamenta.

17th.—Under the influence of digitalis. No pain or palpitation.

Mist. Camph. ℥iss.; Spirit. Ammon. Aromat. ʒss. M. fiat haust. sextis horis sumendus.

From this time she gradually recovered, having taken Cinchona, and had an opium plaster applied to the chest. She was discharged, cured, November 17th.

VII. *Case of Bursal Rheumatism, with metastasis to the head.*

January 9th, 1826.—Thomas ——, (the surgery man at St. George's Hospital.) The ankles and knee-joints are inflamed, and distended with fluid. Pulse 120, soft; skin hot, perspiring; bowels open; tongue white; urine high coloured and scanty. Attacked five days ago with present symptoms.

V.S. ad ℥xij.

Pulv. Ipecac. comp. ℈ss. omni nocte.

Haust. Salin. c. Vini Colchici, ʒss.; Magn. Carbon. ʒss. sextis horis.

Haustus Sennæ, cras mane.

Diæta Lactea.

10th.—Delirious, and complaining of pain in the head. Says he has no pain in the limbs.

V.S. ad ℥xij. Hirudines x. temporibus.
Vespere applicetur Emplastrum Lyttæ nuchæ.
Haustus Sennæ cras mane.

11th.—Is no longer delirious, but complains of severe pain in the head, with intolerance of light, and increased fever. Pain and swelling of joints entirely gone. Pulse full and frequent; tongue furred; skin warm; blood highly inflamed.

V.S. ad ℥xij. Lotio frigida capiti, et repet. V.S. post horas octo nisi prius cesset dolor capitis.—Repetantur alia.

13th.—Head quite relieved, (second bleeding not required on the 11th.) Blood still inflamed. No pain of limbs. Pulse natural; tongue clean; bowels open; urine natural.

Repetantur medicamenta.

16th.—Is now up, and has no complaint.

Decoct. Sarsapar. comp. ℥i. quotidie.
Pulv. Ipecac. comp. ℈ss. omni nocte. Haust. Sennæ, alternis diebus mane.

23d.—Pain and swelling of joints has returned.

Resumatur Haustus Colchici, &c.

25th.—Pain now occupies the sacrum and inner part of the thigh. Joints better. Pulse 100, soft, full; skin warm, moist; tongue white; bowels very open.

V.S. ad ℥xij.

Haustus Salin. c. Vini Colchici, ʒss. sextis horis.

Pulv. Ipecac. comp. ℈ss. omni nocte.—Diæta febrilis.

27th.—No remains of pain, except in the integuments of the chest. Pulse 120, very soft; skin cool and moist; urine natural.

Emplastrum Lyttæ pectori.—Repetantur medicamenta.

From this time he had no complaint but weakness. He took the Sulphate of Quina for some days, and was discharged, cured, on the 13th of February.

